# A Single Center Study of Prescribing and Treatment Outcomes of Patients with Chronic Myeloid Leukemia 

**Published:** 2020-01-01

**Authors:** Ladan Nekoohesh, Mohammad H. Ghahremani, Shahrbano Rostami, Mohsen Nikbakht, Leila Nekoohesh, Roozbeh Naemi, Saeed Mohammadi, Laya Ghadyani Nejad, Seyed Asadollah Mousavi, Mohammad Vaezi, Kamran Alimoghaddam, Bahram Chahardouli

**Affiliations:** 1 Department of Molecular Medicine, School of Advanced Technologies in Medicine, Tehran University of Medical Sciences, Tehran, Iran; 2 Hematology-Oncology and Stem Cell Transplantation Research Center, Tehran University of Medical Sciences, Tehran, Iran; 3 Department of Pharmacology-Toxicology, School of Pharmacy, Tehran University of Medical Sciences, Tehran, Iran; 4 Department of Medical Biotechnology, School of Advanced Technologies in Medicine, International Campus, Tehran University of Medical Sciences, Tehran, Iran; 5 School of Life Sciences and Education, Staffordshire University, Science Centre, Stoke on Trent UK

**Keywords:** Chronic myeloid leukemia, Imatinib, Resistance

## Abstract

**Background:** The present study investigated the patients with Chronic Myeloid Leukemia in chronic phase (CP-CML) who had been on the first- line Imatinib Mesylate (IM) therapy for a period of 84 months.

**Materials and Methods**: This retrospective study was conducted in 295 newly-diagnosed CP-CML patients(age >18 years) who were admitted to the Hematology, Oncology and Stem Cell Transplantation Research Center, Shariati Hospital, Tehran during 1 January, 2009 to 30 December, 2016. Response to treatment was evaluated by molecular response assessment. Rates of IM dose adjustment, switching to another drug therapy, progression to Accelerate Phase (AP) and Blastic Crisis (BC) and long-term outcomes included Overall Survival (OS) and Progression Free Survival (PFS) were assesed.

**Results:** Patients’ average age was 41.7 years, and 52.9% were male. 44.4% of patients at the month 18 achieved Major Molecular Response (MMR). Progression to AP/BC occurred in 26 patients during 84 months, and the estimated rate of OS and PFS were 71.83 and 74.48, respectively. Among the patients who did not achieve MMR at month 18 , 61 patients were treated with IM ( 400 mg /day), and then after month 18, 24(39.3%) of whom achieved MMR. Dose adjustments occurred in 60 patients (20.33%). IM dose increase was observed in 53 patients who did not achive optimal response to imatinib or loss of optimal response. IM dose decrease was observed in 7 patients. 25 (8.47%) patients were switched to a different Tyrosine Kinase Inhibitor (TKI). Most of TKI changes(n=21) happened in patients who did not achieve optimal response to IM and TKI changes owing to adverse events of IM were observed in 4 patients.. Among the patients undergoing change in treatment, 24(43.75%) patients achieved MMR.

**Conclusion: **Our data showed the high effectiveness of the change in the treatment of IM-resistant condition. Moreover, our finding suggests that imatinib be effective in Iranian patients after a long period of time compared to the referenced studies.

## Introduction

 Chronic myeloid leukemia (CML) is a myeloproliferative disease, characterized by the clonal expansion of pluripotent hematopoietic stem cells with reciprocal translocation between chromosomes 9 and 22 (Philadelphia chromosome). This translocation leads to the formation of the BCR-ABL fusion gene that encodes an active tyrosin kinase and plays a crucial role in the pathophysiology of the CML ^1^^, ^^2^ . Most CML patients are diagnosed at the chronic phase and experience mild symptoms. However, the disease can progress to the Accelerated Phases (AP) and Blastic Crisis (BC) which characterized by rising numbers of blast cells in the blood and the bone marrow^   2 ^. CML accounts for 15% of adult’s leukemia and its worldwide incidence is between 1.0 and 1.5 per 100,000^   3 ^. CML occurs in all age groups, but the incidence of it increases by age. CML is more common in males than females with a male/female ratio of 1.2–1.7 3^, ^4 .There is a difference in the median age at diagnosis between developed countries and developing countries. The median age in the developed countries is 57–60 years, whereas the median age in developing countries has been reported 32-44 years^   5 ^. The development of Imatinib Mesylate(IM) and other Tyrosine Kinase Inhibitors (TKIs) for the treatment of CML is associated with improved survival. IM was approved by the FDA in May 2001 as the first line of CP-CML treatment and it is the most effective treatmentin the CP phase 2^,^^4^^,^^6^ . In the International Randomized Study of Interferon and STI571( IRIS), newly diagnosed CP- CML patients treated with IM had an 8-year overall survival (OS) of 83.3%, progression-free survival (PFS) of 93%, and event-free survival (EFS) of 81%. The standard starting dose of IM for the chronic phase is 400 mg/day^7^^,^^8^. Approximately one-third of patients show resistance to IM 7^,^^9^ . In these cases, alternative options include increased IM dose to 600 or 800 mg/day and switching to second and third generation TKIs such as Nilotinib and Dasatinib considered as acceptable approach^4^^,^^6^^,^^10^^,^^11^. 

In this article, we retrospectively analyzed data from 295 Iranian CP- CML patients. The objective was focused on describing some epidemiologic characteristics, treatment patterns and evaluation of response to the treatment and outcomes efficacy.

## MATERIALS AND METHODS

**Study Patients**

This observational retrospective study was conducted using medical records from the Hematology and Oncology Department of Shariati Hospital in Tehran. The samples included 295 newly-diagnosed Philadelphia-positive (PH+)CP- CML patients who were admitted to, treated and followed up in this hospital between 1 January 2010 and 30 September 2016. CP- CML diagnosis was based on European Leukemia Nets (ELN) CML diagnostic criteria^   11 ^ . Adult patients (aged≥18) who received first-line therapy with IM or were treated with Hydroxyurea before IM therapy were eligible to enter the study. Demographics and general characteristics, hematologic analysis, cytogenetical and molecular diagnosis at baseline, molecular assessment at 3, 6, 12, 18 and then at every 6 months, treatment modifications (dose change and drug change)and clinical and laboratory information about disease progression to AP or BC were obtained from medical records.The study was approved by the Ethics Committee of Shariati Hospital.

**Evaluation of response to treatment**

Standardized quantitative real-time reverse transcription-polymerase chain reaction (RT-PCR) method was used to determine BCR-ABL transcript level. The ratio of BCR-ABL to ABL was calculated and reported on the International Scale (IS)^        12 ^. ELN defines the optimal response to treatment as a Major Molecular Response (MMR; BCR-ABL1IS≤ 0.1%) at the month 12 of treatment. Loss of MMR after the month12 is considered as a failure of treatment by ELN. In the current study, the evaluation of response to treatment in patients who did not have molecular assessment at month 12 was determined based on the molecular evaluation at month 18.

**Statistical analysis**

Categorical and numerical variables were described using numbers, frequencies , medians and ranges, respectively. The primary end points of the study were overall survival (OS) and progression-free survival (PFS). OS was defined as the time from the diagnosis until death from any cause. PFS was defined as survival without evidence of accelerated phase (AP) or blast crisis (BC). The Kaplan-Meier method was used to estimate OS and PFS. Statistical analyses were performed using SPSS version 18 software. (SPSS, Chicago, IL, USA).

## Results

**Sample Characteristics**

A total of 295 newly-diagnosed CP-CML patients were enrolled in this retrospective study. Demographic and laboratory characteristics of patients at base line were summarized in Table 1. The median age of patients was 41.7 years. The patient population included 156 (52.9%) men and 139 (47.1%) women. The average length of follow-up was 38 months from the CML diagnosis. All 295 patients received 400 mg/day Indian IM from the beginning of the diagnosis.

**Evaluation of response to treatment up to month 18:**

Up to month 18, among 295CP- CML patients with first –line IM therapy, 4 patients did not refer to the institute for follow-up, 7 patients had progression to the AP or BC, 131 patients obtained MMR and 153 patients did not achieve MMR. Therefore, the rate of patients achieving MMR at month 18 was 44.4% . Among 153 patients who did not achieve MMR at month 18, 61 continued to receive IM ( 400 mg /day), and then after month 18, 24 of whom achieved MMR. 

**Progression to AP/BC and Mortality:**

**Figure 1:**
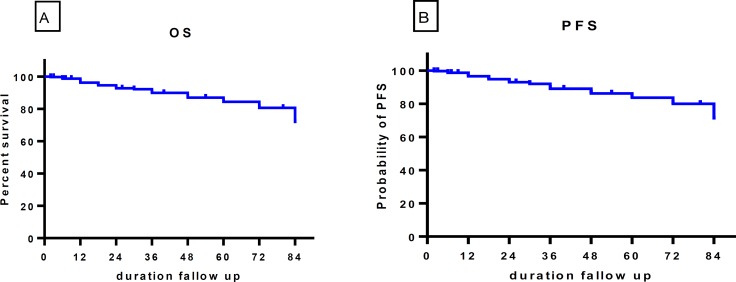
probability of OS (A) and PFS (B) during 84months follow-up

**Figure 2:**
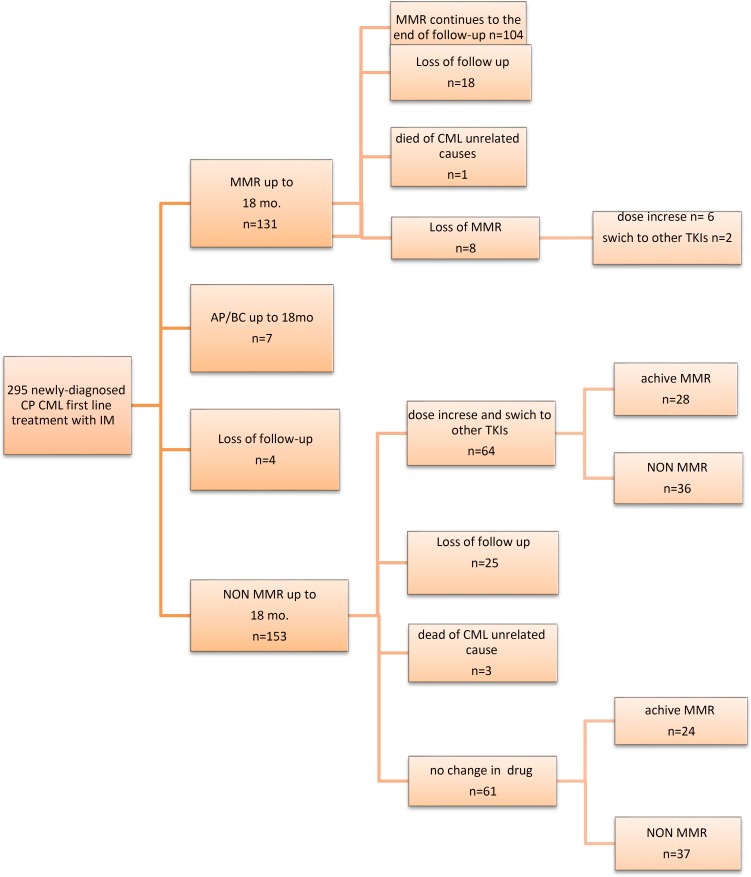
Flow chart showing patient inclusion and follow-up in the study. At first, the assessment was performed until 18th, and then the patients were followed up until the end of treatment. The assessment included achieve MMR, loss of follow up, disease Progression, dose increase and switch to other TKI

Overall, during 84-month follow-up, 26 patients had progression to the AP or BC and 4 patients (1.35%) died of CML unrelated causes. Among 26 AP/BC patients, 24 died before the end of the follow-up period.The estimated OS and PFS were 71.83% and 74.48%, respectively(Figures 1 and 2). The mean and median times to disease progression after diagnosis were 37.8 and 30 months, respectively.

**Therapeutic activity: IM treatment patterns, Study Outcomes and analyses of change in treatment**

Among 153 patients who did not achieve MMR up to month 18 and 8 patients who lost MMR after month 18, 72 received higher dose of IM and were switched to second-generation TKI (Nilotinib).

In 53 patients, IM dose was increased to 600 mg (n=42) or 800 mg (n=11). Out of the 53 patients with dose increase, 7 patients were switched to a different TKI (Nilotinib). Switching to the different TKI occurred in 26 patients. Switching to another TKI was done in patients who did not achieve optimal response to IM (n=22) and in patients who encountered adverse events of IM.

IM dose decrease was observed in 7 patients. Two reasons were responsible for IM dose increase: adverse event (n = 4) and long-term persistent of complete molecular response (n = 3).

The frequency of months to dose increase and decrease and switch from IM to other TKIs are summarized in Table 2 and 3, respectively. 

**Discontinuation of treatment and loss of follow-up**

Up to end of this study, 47 patients did not refere to this center and they were lost to follow-up. Because an uncertain number of patients referred to other medical centers, the rate of patients who discontinued IM was unclear.

**Table 1:** Demographics and disease characteristics at baseline

Sex no. (%) Male Female	156 (52.9%) 139 (47.1%)
Age at CML diagnosis (Year) Mean Median Range	41.7 18-84
Duration of follow-up (Month) Median Range	38 1 – 84
Number of molecular assessment during follow-up Median Range	5.01 2-16
White-cell count Mean ×10^3^/uL Median ×10^3^/uL Range×10^3^/uL Missing data No.	159.506 78 22.5-185 79
Hemoglobin Meangr/dL Mediangr/dL Rangegr/dL Missing data No.	10.59 10 6.7-16.3 79
Platelet count Mean ×10^3^/uL Median ×10^3^/uL Range ×10^3^/uL Missing data No.	391.71 310 20-2560 79
Peripheral blood blasts Median Range Missing data No.	1 0-6 79
Number of molecular assessment during follow-up Median Range	4.9 2.16

**Table 2:** The frequency of months for dose adjustment

**Dose adjustments**	**No.**
Dose increase Dose increase from IM initiation to 600 mg/day Dose increase from IM initiation to 800 mg/day	53 42 11
Causes of increasing doses Non Optimal Response Loss Of Response (relapse)	45 8
Months to dose increase from IM initiation After 12 or 18 months from IM initiation (no) After 24 months from IM initiation (no) After36 months from IM initiation (no) After48 months from IM initiation (no)	21 19 7 6
Dose decrease dose decrease from 400 mg/day to 300 dose decrease from 400 mg/day to 200	7 5 2
Causes of decreasing doses Dose decrease due to complication of imatinib therapy Dose decrease due to long-term persistent complete molecular response	4 3

**Table 3:** The frequency of months to switch to other TKLs

switching to other TKIs (Nilotinib)	NO.
**Switching to other therapy after dose increase**	7
**Switching to other therapy without dose increase** **After 12 or 18 months from IM initiation (no)** **After 24 months from IM initiation (no)** **After36 months from IM initiation (no)** **After48 months from IM initiation (no)**	19 8 5 2 4

## Discussion

 This retrospective observational study described treatment patterns and disease outcomes in 295 Iranian adult CP-CML PH+ patients. The mean age of the patients at the time of diagnosis was 41.7 years .This is similar to the results of studies in developing countries that reported the median age at diagnosis between 32 and 44 years    5 . Moreover, in the current study, the male to female ratio in CML patients was 1.2 and this is in line with other studies that reported this ratio between 1.2 and 1.7 3^,^^4^ .

In the present study, all patients received a dose of 400 mg Indian IM per day as the first-line therapeutic regimen. According to ELN and NCCN recommendation, patients who do not achieve MMR or CCyR at month12 (failure to first-line IM therapy ) may receive a dose escalation or switch to second and third generation TKIs^11^^, ^^13^^, ^^14^. In the current study, only molecular assessment was carried out. Furthermore, the evaluation of response to treatment in patients, who did not undergo molecular assessment at month12, was determined based on the molecular evaluation at month 18. Up to month 18, 131 out of 295 patients achieved MMR . The rate of patients achieving MMR at month 18 was lower compared to referenced clinical trials (44.4% in our stuy compared to 64.8% in IRIS study)^   7 ^. The low molecular response rate in our study can be due to low adherence of patients to medication, difference of drug brand and difference in response to IM treatment. 

In this study, the estimated overall survival rate and progression-free survival with first-line imatinib therapy at month 84 was 71.83% and 74.48%, respectively, whereas in the international randomized study of interferon and STI (IRIS), the 6-year OS and PFS were reported to be 88% and 93%, respectively and the estimated OS rate at 11 years with first-line imatinib therapy was 83.3% 7^, ^8 .

According to ELN recommendations, patients who do not achieve MMR at 12 months are considered as failures to IM treatment ^4^^,^^6^^,^^13^ . Switching to second-line TKIs and dose escalation of IM can increase response rates in patients with inadequate response to IM ^15^^-^^18^ . In this study, among the patients, who did not achieve MMR at month 12 or 18, 64 patients received dose-escalated IM and were switched to second-line TKIs. However, it is important to note that most of the patients were diagnosed with CML before 2013, and the change in the treatment of patients was more common in patients who were diagnosed with CML after 2013. Our data showed that among the patients undergoing change in treatment, 24(43.75%) achieved MMR.This rate indicates the high effectiveness of the treatment change in IM-resistant patients. Among 153 patients who did not achieve MMR at month 18, 61 continued to receive IM ( 400 mg /day), and then after month 18, 24 of whom achieved MMR. This information suggests that imatinib be effective in Iranian patients after a long period of time compared to referenced studies  ^6^^, ^^19^ . 

## Limitations

Similar to other retrospective studies, there were some limitations to our study. First, there was not data available to assess spleen size for numerous of the patients, so we could not obtain Sokal score to predict prognosis of the disease. Moreover, there were no hematological data in a large number of patients. Second, the response to imatinib treatment was evaluated through molecular response evaluation and there was no cytogenetic evaluation in vast majority of patients; therefor, we assessed response to imatinib only based on molecular response evaluation. Third, about 40% of patients did not continue to follow imatinib therapy at this center, so there is no information about their outcomes. Finally, the study was based on data from a single center, although this center is a referral center.
